# ﻿Genital anatomy, jaw and radula of *Guladentiasubtussulcata* (Helicoidea, Cepolidae), endemic to western Cuba

**DOI:** 10.3897/zookeys.1080.73194

**Published:** 2022-01-04

**Authors:** Maike Hernández1, Manuel A. Bauzá1, Thierry Backeljau2,3

**Affiliations:** 1 Institute of Ecology and Systematics, Carretera Varona Km 3 1/5, Capdevila, Havana, Cuba Institute of Ecology and Systematics Havana Cuba; 2 Royal Belgian Institute of Natural Sciences (RBINS), Vautierstraat 29, B-1000 Brussels, Belgium Royal Belgian Institute of Natural Sciences (RBINS) Brussels Belgium; 3 Evolutionary Ecology Group, University of Antwerp, Universiteitsplein 1, B-2610 Antwerp, Belgium University of Antwerp Antwerp Belgium

**Keywords:** Auxiliary copulatory organ, reproductive system, sheath-like accessory gland, Stylommatophora, West Indies

## Abstract

This study provides the first data on the genital anatomy, jaw and radula of *Guladentiasubtussulcata* (L. Pfeiffer, 1863). The auxiliary copulatory organ of this species is very peculiar, similar to that of *Jeanneretia* L. Pfeiffer, 1877, and different from that of other cepolids. It consists of an elongate, pedunculate mucus gland inserted apically on a muscular papilla and an atrial sac, all covered by a sheath. A sheath-like accessory gland is inserted at the base of the atrial sac. Another similarity with *Jeanneretia* is the presence of a fertilization pouch-spermatheca complex with a single exposed spermatheca. Like *Jeanneretia*, *G.subtussulcata* has an oxygnath, highly arched jaw with slight striae over the entire surface and a broad, well-developed median projection. The radula has triangular and monocuspid central and lateral teeth (the central teeth are smaller than the rest). The marginal teeth are multicuspid with the mesocone and ectocones smaller than the endocones. The similar structures of the auxiliary copulatory organ (without dart sac) and spermatheca (simple) strongly suggest that *G.subtussulcata* and *Jeanneretia* spp. are closely related. As such, it remains to be decided whether *Guladentia* Clench & Aguayo, 1951 and *Jeanneretia* should continue to be treated as separate genera.

## ﻿Introduction

**Figure 1.**
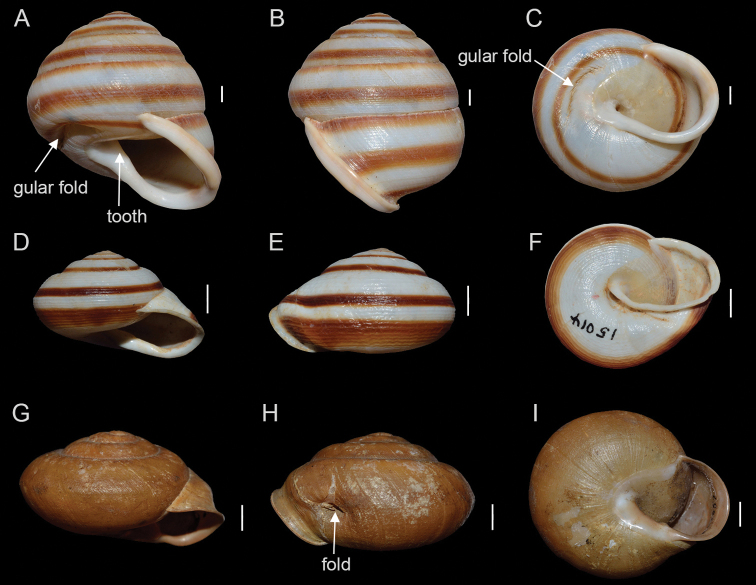
Shells of *Guladentiasubtussulcata***A–C** (**A** and **B** lateral views and **C** ventral view), *Jeanneretiaparraiana***D–F** (**D** and **E** lateral views and **F** ventral view), *Cepoliscepa***G–I** (**G** and **H** lateral views and **I** ventral view). Scale bar = 5 mm.

*Guladentia* Clench & Aguayo, 1951, is a Cuban endemic terrestrial snail genus of the family Cepolidae. It was originally described as subgenus of *Jeanneretia* L. Pfeiffer, 1877 (Clench and Aguayo 1951) with *Helixsubtussulcata* L. Pfeiffer, 1863 as type species and comprising three other species, viz. *Cepolistorrei* Clench & Aguayo, 1933, Jeanneretia (Guladentia) modica Clench & Aguayo, 1951 and J. (G.) gundlachi Clench & Aguayo, 1951. While *Guladentia* was explicitly maintained as a subgenus by Espinosa and Ortea (1999, 2009) and implicitly by Fontenla et al. (2013), it was considered as a separate genus by Zilch (1960), Schileyko (2004) and Hernández et al. (2017), though without providing a rationale. As such, *Guladentia* differs conchologically from *Jeanneretia* by (1) possessing a shell with a well-defined “gular” fold, which is an invagination of the shell on the base of the body whorl, curved and parallel with the whorl about midway between the whorl periphery and the columella, and by (2) having a long and curved tooth on the base of the shell within the aperture. Thus, according to Clench and Aguayo (1951) the shell structure of *Guladentia* would resemble that of the genus *Cepolis* Montfort, 1810 (Fig. 1).

Species of *Guladentia* occur in mountainous limestone areas in the central-western region of the Sierra de los Órganos, Cuba. They are quite rare (Clench and Aguayo 1951), yet, *G.subtussulcata* is the species with the widest distribution and that is the least difficult to collect. It is also the largest species in the genus (shell height: 16–31 mm; shell diameter: 21–31 mm).

Hernández et al. (2020) emphasized that the genital anatomy of a majority of Cepolidae taxa, including the genus *Guladentia*, is still unknown. The present contribution, therefore, aims to provide the very first data on the genital anatomy, jaw, and radula of the type species of this genus, viz. *G.subtussulcata*, and on the basis of these new data it also briefly reflects on the (sub)generic status of *Guladentia*.

## ﻿Materials and methods

Nine live specimens of *G.subtussulcata* were collected from three localities in Viñales, Pinar del Río Province, Cuba. The material examined is deposited in the Malacological Collection of the Institute of Ecology and Systematics (CZACC; Colección Zoológica de la Academia de Ciencias de Cuba).

**Material examined** • 7 specimens; Mogote El Valle; 22°37'9.3"N, 83°41'33.09"W; 20 May 2014; M. Hernández Leg.; CZACC8. A.0300 to 0307 • 1 specimen; Mogote Dos Hermanas; 22°37'05"N, 83°44'38"W; 5 July 2014; M. Hernández Leg.; CZACC8. A.0308 • 1 specimen; Sierra de Viñales; 22°38'36"N, 83°44'46"W; 6 July 2014; M. Hernández Leg.; CZACC8. A.0309.

Specimens were drowned for 12 h in an airtight jar filled with water, after which they were removed from their shells and fixed in 70% ethanol. Specimens were dissected using a Carl Zeiss Stemi 2000 stereomicroscope. The reproductive tracts were photographed with a Nikon 5100 camera. Radula and jaw were extracted manually, cleaned by soaking in 10% KOH solution for about 6 h followed by rinsing in ethanol. They were mounted for scanning electron microscopy with a Thermo Fisher Quanta 200 scanning electron microscope.

The terminology of the reproductive apparatus follows Baur (2010) and Hernández et al. (2020). The length of seven genital structures was measured using scale paper (error 1 mm): flagellum, penis + distal epiphallus, proximal epiphallus, bursa-copulatrix duct, auxiliary copulatory organ, and length of the spermoviduct.

## ﻿Results

### ﻿Genital anatomy

**Figure 2.**
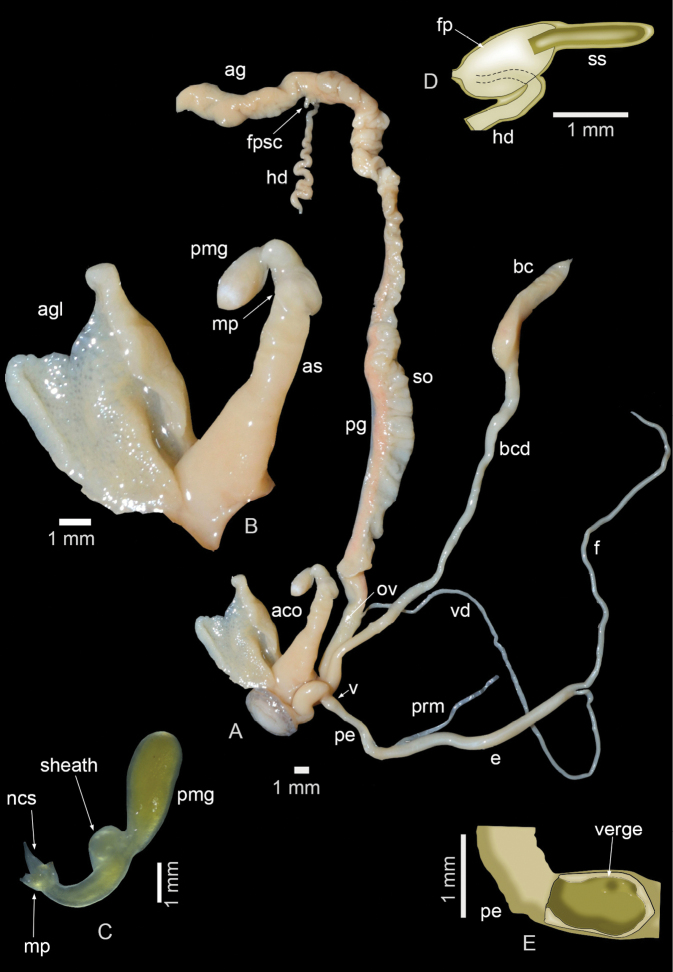
Genital anatomy of *Guladentiasubtussulcata***A** genitalia **B** auxiliary copulatory organ details) **C** details of the distal portion of the auxiliary copulatory organ **D** drawing of the fertilization pouch–spermathecal complex **E** drawing of the details of the verge. Abbreviations: aco = auxiliary copulatory organ, ag = albumen gland, agl = accessory gland, as = atrial sac, bc = bursa copulatrix, bcd = bursa-copulatrix duct, e = proximal epiphallus, fl = flagellum, fp = fertilization pouch, fpsc = fertilization pouch–spermathecal complex, hd = hermaphroditic duct, mp = muscular papilla, ncs = non-calcareous structure, pe = penis + distal epiphallus, ov = oviduct, pmg = pedunculated mucus gland, pg = prostatic gland, prm = penial retractor muscle, so = spermoviduct, ss = single spermatheca, v = verge, vd = vas deferens.

The complete genitalia is shown in Fig. 2A. The fertilization pouch–spermathecal complex comprises a fertilization pouch embedded in the albumen gland and a single, exposed spermatheca (Fig. 2D). Spermoviduct very long (mean length: 57 mm; range: 42–79 mm), in length followed by the bursa-copulatrix duct (mean length: 46 mm; range: 23–78 mm) and the flagellum (mean length: 39 mm; range: 23–65 mm). Vagina short. The penis + distal epiphallus of medium length (mean length: 8 mm; range: 5–14 mm), thin and cylindrical, internally with a verge located in the first third, which may be swollen and somewhat wrinkled (Fig. 2E). Proximal epiphallus (mean length; 15 mm; range: 7–26 mm) is longer than the penis + distal epiphallus. The auxiliary copulatory organ (mean length: 17 mm; range: 11–20 mm) (Fig. 2B, C) is soft and has no dart sac. It consists of an elongate, pedunculated mucus gland inserted apically on a muscular papilla (Fig. 2C) and an atrial sac (Fig. 2B), all covered by a sheath. One sheath-like accessory gland (consisting of alveoli) is inserted at the base of the atrial sac (Fig. 2B).

### ﻿Jaw and radular morphology

**Figure 3.**
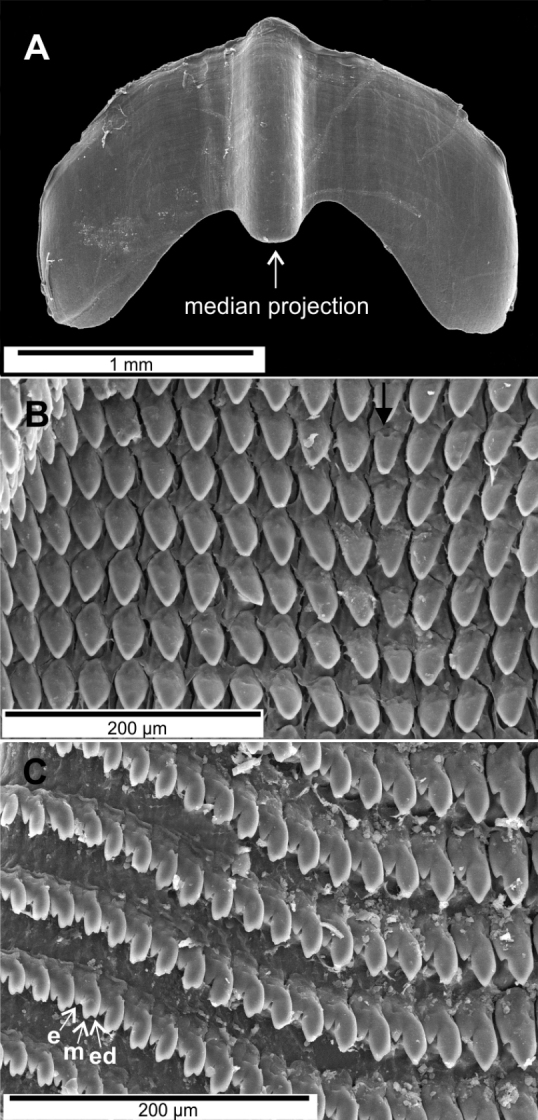
Scanning Electron Microscope photographs of the jaw and radula of *Guladentiasubtussulcata*. **A** jaw **B** central and lateral teeth, central teeth marked with a black arrow **C** transitional and marginal teeth, the latter with their ectocones (e), mesocones (m), and endocones (ed).

The jaw is oxygnath (Fig. 3A), solid, high arched, almost smooth except for slight striae all over the surface, and with a wide, well-developed, median projection. The radula has a monocuspid, pointed and triangular central tooth, which is smaller than the other teeth and shorter than the base of the tooth (Fig. 3B). Lateral teeth monocuspid, pointed, triangular and as long as their base (Fig. 3B). Between the lateral and marginal teeth, there are transitional teeth with ectocones. Marginal teeth multicuspid with the mesocone and ectocones smaller than the endocones (Fig. 3C).

## ﻿Discussion

The reproductive system of *Guladentiasubtussulcata* is similar to that of other cepolid species by, amongst other features, the presence of an auxiliary copulatory organ. Yet, while in other cepolids this auxiliary copulatory organ contains a dart sac (on top of the muscular papilla), there is no dart sac in *G.subtussulcata* (Fig. 2B) and *Jeanneretia* ssp. (Hernández et al. 2021). Instead, these latter two taxa have a non-calcareous ‘dart’ (Fig. 2C), which is fused to a muscular papilla that opens directly into the atrial sac. More generally, the auxiliary copulatory organ of *G.subtussulcata* is similar in size and structure to that of *Jeanneretia* spp. as described by Hernández et al. (2021). In this sense it represents a unique feature within Cepolidae. Also, the simple spermatheca of *G.subtussulcata* is a unique feature within Cepolidae that links this species with *Jeanneretia* spp.

Conversely, *G.subtussulcata* and *Jeanneretia* ssp. differ markedly by their accessory glands: *G.subtussulcata* has a single sheath-like accessory gland (Fig. 2B), a unique feature within Cepolidae, whereas *Jeanneretia* ssp. have a pair of tubular accessory glands. Furthermore, compared to the data provided for *Jeanneretia* spp. by Hernández et al. (2021), *G.subtussulcata* has a much longer spermoviduct, but a much shorter flagellum, than *Jeanneretia* spp.

The jaw and radula of *G.subtussulcata* are extremely arched (Fig. 3A) like in *Jeanneretia* ssp. (Hernández et al. 2021), though the radular teeth of *G.subtussulcata* are a little more pointed than in *Jeanneretia* spp. In general, the radular morphology of *G.subtussulcata* complies with that of other cepolid genera, except *Polymita* Beck, 1937 (Moreno 1950), in which the radula is arranged in a “v” shape with all teeth equal in size (Hernández et al. 2021).

The structure of the auxiliary copulatory organ (without dart sac) and the spermatheca (simple) strongly suggests that *G.subtussulcata* and *Jeanneretia* spp. are closely related. Hence, the question arises whether *Guladentia* should be regarded as a separate genus or as a subgenus of *Jeanneretia*. The conchological differences between *G.subtussulcata* and *Jeanneretia* spp. may indicate a separation at the genus level, with the gular shell fold (Fig. 1A) and the unique accessory gland structure (Fig. 2B) of *Guladentia* as the defining features of the genus. However, the genital anatomy of more *Guladentia* species should be studied and combined with DNA sequence data to infer the phylogenetic relationships of *Guladentia* and *Jeanneretia*. These additional data are not only necessary to verify whether these two taxa are monophyletic, but also to explore whether the conchological similarities between *Guladentia* and *Cepolis* suggested by Clench and Aguayo (1951) are synapomorphic or homoplasic character states.
